# Factor V-Leiden Mutation: A Common Risk Factor for Venous Thrombosis among Lebanese Patients

**DOI:** 10.1155/2012/380681

**Published:** 2012-06-12

**Authors:** Raghid Kreidy

**Affiliations:** Department of Vascular Surgery, Saint George Hospital, University Medical Center, University of Balamand, P.O. Box 166378, Achrafieh, Beirut 1100 2807, Lebanon

## Abstract

*Aim*. Lebanon exhibits one of the highest prevalences of factor V-Leiden (FVL) in the world (14.4%). The aim of this study is to evaluate the incidence of FVL mutation among Lebanese patients with lower extremity venous thrombosis. *Material and Methods*. From January 2003 to January 2011, 283 consecutive Lebanese patients, diagnosed with deep venous thrombosis (DVT) by duplex scan, were retrospectively reviewed. FVL mutation was tested among patients with conditions highly suggestive of hypercoagulation states (65 patients). *Results*. FVL mutation was detected among 56.9% of patients, 68.6% of patients younger than 50 years, and 43.4% of patients older than 50 years (*P* = 0.041). FVL mutation was commonly reported in young adults, in patients with pregnancy, estrogen drugs, recurrent DVT, and resistance to anticoagulation. *Conclusion*. The high rate of FVL mutation observed among Lebanese patients with venous thrombosis is related to the high prevalence of this mutation in the Lebanese population. Thrombophilia screening should be tailored to accommodate a population's risk factor. In countries with high prevalence of FVL, this mutation should be screened among patients younger than 50 years and patients with situations highly suggestive of hypercoagulation states.

## 1. Introduction

 The appropriate management of venous thrombosis requires a thorough knowledge of diagnostic and treatment modalities. However, an understanding of the underlying epidemiology and associated risk factors is equally essential. Pathophysiology of venous thrombosis is multi-factorial and involves environmental, acquired, and genetic factors. Factor V R 506 Q-Leiden is the most frequent prothrombotic genetic abnormality leading to thrombophilia. The prevalence rate of this mutation varies according to ethnic and geographic distribution of the populations. The allele mutation is high among Eastern Mediterranean populations and extremely high among Lebanese population. The aim of this study is to determine the incidence of factor V-Leiden mutation among Lebanese patients with lower extremity venous thrombosis and high risk situations for hypercoagulation states and to discuss the reported results according to epidemiologic and pathophysiologic publications in the international literature.

## 2. Material and Methods

 From January 2003 to January 2010, 283 consecutive Lebanese patients (115 males and 168 females), diagnosed in an academic tertiary-care center with lower extremity deep venous thrombosis using color flow duplex scan (Prosound Alpha 7 ALOKA, Zug, Switzerland), were retrospectively reviewed. Age varied between 21 to 96 years (mean: 64.9 years). Factor V-Leiden was screened among 65 of the 283 patients, presenting with specific conditions highly suggestive of hypercoagulation states according to the guidelines proposed by the European Genetics Foundation, the Cardiovascular Educational and Research Trust, the International Union of Angiology, and the Mediterranean League on Thromboembolism [1]. These conditions were young age, pregnancy, oral contraceptives and estroprogestative drugs, spontaneous, extended and recurrent DVT, family history, resistance to anticoagulation, long-haul air travel, and constituted the inclusion criteria for testing FVL.

There was no exclusion criteria. Accordingly, FVL mutation was tested in 37 young patients, in 9 pregnant patients, in 5 patients using oral contraceptives or treated with hormones, in 32 patients with spontaneous venous thrombosis, in 9 patients with extended venous thrombosis, in 15 patients with recurrent venous thrombosis, in 11 patients with family history of venous thromboembolism (VTE), in 4 patients with resistance to anticoagulants, and in 2 patients with long-haul air travel. Fourteen patients presented with one of these high risk conditions requiring FVL testing, 18 with two, 2 with three and 3 with four conditions.

Inherited prothrombotic risk factors including FVL, prothrombin G 20210 A, factor V H 1299 R, MTHFR C 677 T, and MTHFR A 1298 C mutations were determined using CVD strips according to the protocol supplied by the manufacturer (CVD Strip Assays R, ViennaLab, Austria). Plasma homocysteine level was also reported.

Patients were subdivided into 2 groups according to age—A: younger than 50 years (35 patients) and B: older than 50 years (30 patients).

A clinical research form was filled out with every patient, by a retrospective evaluation of clinical presentation and risk factors for venous thrombosis; data were entered and analysed by SPSS Statistics (IBM Corporation, Somers, NY, USA) software, version 13.0. A chi-square test was used to correlate between dichotomous variables, and a Fisher exact test was used in case of calculated values that were lower than five.

## 3. Results

Factor V-Leiden was detected among 37 patients representing 56.9% of the tested patients. More than one clinical risk factor was associated with a higher risk of positivity for FVL (63% comparing to 44% for the other prothrombotic genetic mutations). Five patients (7.7%) were carriers for the homozygous form and thirty-two (49.2%) for the heterozygous form. Factor V-Leiden was isolated in 19 patients and associated with prothrombin G 20210 A mutation in 4 patients and with other prothrombotic genetic mutations in 14 patients (MTHFR C 677 T mutation: homozygote: 2, heterozygote: 9, MTHFR A 1298 C mutation: heterozygote: 8, and V H 1299 R mutation: heterozygote: 1) (Table 1). Plasma homocysteine level was increased in 8 patients. The association with other genetic risks did not increase the risk of recurrent DVT but was determinant of more severe and extended forms of VTE. Factor V-Leiden was more commonly reported in the younger age group 9 (group A) comparing to the older age group (group B) (68.6% comparing to 43.4%; *P* = 0.041). 

 FVL mutation was detected among 66.6% of pregnant patients, 60% of patients using oral contraceptives or treated with hormone therapy, 56.2% of patients with spontaneous DVT, 55.5% with extended DVT, 66.6% of patients with recurrent DVT, 45.4% of patients with family history of venous thromboembolism, 100 % of patients with resistance to anticoagulants and 50% of patients with long-haul air travel (Table 2).

## 4. Discussion

Resistance to activated protein C degradation caused by a mutation in the factor V R 506 Q-Leiden is the most prevalent inherited cause of venous thrombosis. The prevalence rate of factor V-Leiden in the general population varies from 0% to 15% according to ethnicity and geographic distribution [2, 3]. The allele mutation is low in African, Asian, South European populations (1%–3%), relatively high in United States populations (5%) and high in Mediterranean populations (13.6% in Syria, 12.3 % in Jordan, and 13.4% in Greece) [4, 5]. Lebanon exhibits one of the highest frequencies of FVL mutation in the Eastern Mediterranean and in the world with a prevalence of 14.4% in the general population [6].

Among the inherited thrombophilias, factor V-Leiden gene mutation is the most common predisposing factor, accounting for 10% to 20% of VTE in large population studies [7, 8]. Some authors suggest that this mutation is present in up to 37% of patients with postthrombotic syndrome [1]. The lifetime probability of symptomatic VTE in patients with heterozygous FVL mutation is approximately 10% [9]. The risk of VTE is increased 3- to 7-fold in heterozygotes for FVL and 50- to 100-fold in homozygotes [8, 9].

The prevalence of FVL mutation among patients with venous thrombosis varies considerably according to the origin of the reported study. It is extremely low in Singapore (0.26%), low in Spain and in the United States (8.3% and 9%, resp.), relatively high in the United Kingdom, Greece, and Tunisia (15.6%, 16.8%, and 20.3%, resp.) and high in Italy (24.3%) [10–15]. This prevalence significantly increases among adults younger than 50 years of age in Denmark (32.7%) and Czech Republic (40%), but remains very low in India (3%) [16–18]. Children developing venous thrombosis are often carriers for FVL mutation. Unal et al. suggested that homozygosity for FVL leads to the development of thrombosis at a young age [19]. Aschka et al. demonstrated that 26% of children less than 5 years of age and 30% of children more than 10 years of age with venous thrombosis and 29% of children with spontaneous venous thrombosis have harbored FVL mutation [20]. In these series, the high rate of factor V-Leiden mutation observed among the tested patients (56.9%) and the tested young adults (group A) (68.6%) is related to the high prevalence of this mutation in the Lebanese population. The mutant allele was highly expressed among Lebanese patients with DVT and high risk conditions for hypercoagulation states. These findings confirm that FVL mutation is a very common risk factor for venous thrombosis among Lebanese patients and especially young Lebanese adults with high risk situations, emphasizing the importance of screening for this mutation in this particular group of patients.

VTE recurrence rate increases significantly in adults and children when venous thrombosis is associated with primary hypercoagulopathy and essentially with factor V-Leiden mutation [21, 22]. More than 66% of the reported patients with recurrence were carriers for FVL mutation.

FVL mutation, especially in its homozygote form, increases the risk for venous thrombosis 34-fold among pregnant women and 35-fold among contraceptive users [13, 23–25]. FVL mutation has been identified in 33.3% of pregnant women and 6.5% of oral contraceptive users women [26, 27]. In these series, a high percentage of pregnant women (66.6%) and of women treated with estrogen drugs (60%) were carriers for FVL mutation. Screening for FVL mutation among Lebanese pregnant women and among women treated with estrogen and presenting for venous thrombosis is warranted.

 A very high incidence of FVL mutation was particularly detected among patients manifesting resistance to anticoagulants (100%). Testing for FVL among Lebanese patients is highly recommended when venous thrombosis is extensive and progressive despite adequate anticoagulation.

FVL mutation interacts with other concurrent acquired and thrombophilic conditions such as cancer, surgery, long-haul air travel, and with associated prothrombotic genetic abnormalities and essentially prothrombin G 20210A mutation to increase the risk of incident venous thrombosis [9, 28–33]. The relative risk for venous thrombosis is calculated to be 2-3-fold for the prothrombin mutation alone and 20-fold for a combination with FVL mutation [34]. The three reported patients harboring this combination were young adults and two of them presented for extensive and bilateral VTE. FVL acts also as a concurrent risk factor in individuals with other prothrombotic polymorphism and hyperhomocysteinemia leading to a synergistic gene-gene interaction often increasing the risk above the sum of individual risk factors [35].

In these series, FVL mutation was associated with MTHFR C 677 T mutation in 9 patients, MTHFR A 1298 C mutation in 8 patients and VH 1299 R mutation in 1 patient. Of the six reported patients carriers for two or more prothrombotic genetic defects in addition to FVL mutation and presenting increased homocysteine level, four patients manifested bilateral extended DVT with pulmonary embolism resistant to anticoagulants and requiring inferior vena cava filter insertion.

Duration of treatment for venous thrombosis among patients carriers for FVL mutation depends on the nature and extension of the thrombosis, the underlying risk factors, the presence of a previous VTE, and the presence and the severity of concurrent prothrombotic genetic mutation. Patients homozygotes for FVL mutation and double heterozygotes for FVL and prothrombin mutation are considered for extended long-term treatment with anticoagulants therapy [36].

## 5. Conclusion

The high rate of factor V-Leiden mutation observed among Lebanese patients with lower extremity venous thrombosis is related to the high prevalence of this mutation among the Lebanese population. This factor interacts with other concurrent acquired and thrombophilic conditions to increase the risk of incident venous thrombosis.

The prevalence of FVL mutation is particularly increased among young patients, pregnant patients, patients treated with estrogen, patients with recurrent DVT, and patients with resistance to anticoagulation. The association of two or more prothrombotic genetic defects to FVL mutation further increases the risks and the severity of venous thrombosis.

Thrombophilia screening should be tailored to accommodate a population's risk factor. In countries with high prevalence of factor V-Leiden, this mutation should be tested among patients younger than 50 years even with a transient risk factor and patients with situations highly suggestive of hypercoagulation states. This permits to extend the duration of anticoagulant therapy in high risk patients reducing the incidence of postthrombotic syndrome and recurrent venous thromboembolism.

## Figures and Tables

**Table 1:** Prothrombotic genetic mutations associated with FVL mutation.

Prothrombotic genetic mutation	Number of patients
Prothrombin G 20210 A	4
MTHFR C 677 T	11
MTHFR A 1298 C	8
V H 1299 R	1

**Table 2:** Conditions associated with FVL mutation.

High risk conditions	Patients screened for FVL mutation	Patients carriers for FVL mutation
Young patients (A group)	35	24 (68.6%)
Pregnancy	9	6 (66.6%)
Estrogen treatment	5	3 (60%)
Spontaneous DVT	32	18 (56.2%)
Extended DVT	9	5 (55.5%)
Recurrent DVT	15	10 (66.6%)
Family history of VTE	11	5 (45.4%)
Resistance to anticoagulant	4	4 (100%)
Long-haul air travel	2	1 (50%)
